# The *yapA* Encodes bZIP Transcription Factor Involved in Stress Tolerance in Pathogenic Fungus *Talaromyces marneffei*

**DOI:** 10.1371/journal.pone.0163778

**Published:** 2016-10-05

**Authors:** Wiyada Dankai, Monsicha Pongpom, Sirida Youngchim, Chester R. Cooper, Nongnuch Vanittanakom

**Affiliations:** 1 Department of Microbiology, Faculty of Medicine, Chiang Mai University, Chiang Mai, Thailand; 2 Center for Applied Chemical Biology and Department of Biological Sciences, Youngstown State University, One University Plaza, Youngstown, OH, 44555, United States of America; Institute of Microbiology, SWITZERLAND

## Abstract

*Talaromyces marneffei*, formerly *Penicillium marneffei*, is a thermally dimorphic fungus. It causes a fatal disseminated disease in patients infected with the human immunodeficiency virus (HIV). Studies on the stress defense mechanism of *T*. *marneffei* can lead to a better understanding of the pathogenicity and the progression of the disease due to this fungus. The basic leucine-zipper (bZip) transcription factor gene in *Saccharomyces cerevisiae*, named *yap1* (yeast activating protein-1), is known as a crucial central regulator of stress responses including those caused by oxidative agents, cadmium, and drugs. An ortholog of *yap1*, designated *yapA*, was identified in *T*. *marneffei*. We found that the *yapA* gene was involved in growth and fungal cell development. The *yapA* deletion mutant exhibited delays in the rate of growth, germination, and conidiation. Surprisingly, the *yapA* gene was also involved in the pigmentation of *T*. *marneffei*. Moreover, the mutant was sensitive to oxidative stressors such as H_2_O_2_ and menadione, similar to *S*. *cerevisiae yap1* mutant, as well as the nitrosative stressor NaNO_2_. In addition, the *yapA* mutant demonstrated significantly decreased survival in human macrophage THP-1 compared to wild-type and complemented strains. This study reveals the role of *yapA* in fungal growth, cell development, stress response, and potential virulence in *T*. *marneffei*.

## Introduction

*Talaromyces marneffei*, formerly known as *Penicillium marneffei*, is a temperature-dependent dimorphic fungus. It causes a fatal disseminated disease in immunocompromised hosts, especially HIV-infected patients. A high incidence of *T*. *marneffei* infection occurs in Southeast Asia, particularly Thailand, Northeastern India, Hong Kong, Southern China, Vietnam, and Taiwan [1]. Moreover, there were cases of *T*. *marneffei* infection from Ghana and Togo who reportedly had never been in Asia [2, 3]. The *T*. *marneffei* infection was also reported in non-HIV-infected individuals, including patients with hyper IgE syndrome [4], x-link hyper IgM disease [5], systemic lupus erythematosus [6], and patients with impaired INF-γ and T_H_17 response [7]. Once the conidia are inhaled into host lungs, macrophages phagocytose and destroy the conidia. However, disseminated infections occur frequently in immunodeficiency patients when the inhaled conidia convert into yeast cells within macrophages and spread to other organs [1, 8]. Resistance to stresses induced by the reactive oxygen species produced by phagocytes is one potential key to intracellular survival of fungi and subsequent dissemination.

To understand the pathogenesis of *T*. *marneffei*, more studies of the fungal determinants associated with virulence are needed. Some of the genes involved in oxidative stress response in *T*. *marneffei* have been identified, such as those encoding catalase-peroxidase [9] and copper, zinc superoxide dismutase [10]. In addition, Cao and collaborators [11] demonstrated that the complementation of a *Saccharomyces cerevisiae skn7* disruptant strain by the *T*. *marneffei skn*7 transcription factor contributed to the oxidative stress response of the former species. This observation suggested that the oxidative stress response in *T*. *marneffei* was also related to the *skn7* gene. However, the mechanism of survival of *T*. *marneffei* under oxidative stress remains unclear. Studies on the stress defense mechanism of *T*. *marneffei* can lead to a better understanding of the pathogenicity and the progression of the disease due to this fungus.

The basic leucine-zipper (bZip) transcription factor gene *yap1* (yeast activating protein-1) in *S*. *cerevisiae* is known as a crucial central regulator of stress response including that caused by oxidative agents, cadmium, and drugs [12]. Loss of *yap1* function resulted in hypersensitivity to superoxide anion radical. The Δ*yap1* strain has reduced specific activities of several enzymes involved in oxygen detoxification such as superoxide dismutase, glucose-6-phosphate dehydrogenase, and glutathione reductase [13]. The *yap1*-like homologs have been identified in other organisms. For example, *pap1*^+^ in *Schizosaccharomyces pombe* encodes an AP-1-like transcription factor that contains a region rich in basic amino acids followed by a leucine zipper motif [14]. Similar to the Yap1p in *S*. *cerevisiae*, Pap1p appears to be involved in the transcriptional regulation of some anti-oxidant genes in response to oxidative stress [15].

Among some pathogenic fungi, the bZip transcription factor in *Candida albicans*, Cap1 protein (Cap1p), which is homologous to Yap1p, is involved in oxidative stress responses and multidrug resistance [16]. In the filamentous fungus, *Aspergillus fumigatus*, the *Afyap1* gene regulates several defense genes against oxidative stress [17].

As mentioned above, the *yap1* gene of *S*. *cerevisiae* has been involved in oxidative, metal, and drug stress responses. Therefore, investigations pertaining to the role of *T*. *marneffei* orthologue, *yapA*, against environmental stress should provide a means to understand the pathogenicity in *T*. *marneffei*. Functional analysis of the *yapA* gene using gene deletion and complementation analysis may provide important data to explain the mechanism of intracellular pathogenicity of *T*. *marneffei*. In this report, we present data that demonstrated effect of the *yapA* gene upon radial growth, conidial development, pigmentation, processing of fission division, and stress response in *T*. *marneffei*. Moreover, our studies found that the *yapA* might be a virulence factor of *T*. *marneffei* by improving the ability of this fungus to survive in host immune cells.

## Materials and Methods

### Strains and culture conditions

*T*. *marneffei* uracil auxotroph strain G816 (Δ*ligD niaD pyrG*^-^), a strain that facilitates efficient homologous integration, and strain G809 (Δ*ligD*::*pyrG*^+^ *niaD pyrG*) genetically modified from *Talaromyces marneffei* FRR2161 (CBS 334.59, ATCC18224) [18] were provided by Dr. Alex Andrianopoulos (Department of Genetics, The University of Melbourne, Parkville, Victoria, Australia). The *T*. *marneffei* G816 strain was maintained on *Aspergillus nidulans* synthetic medium (ANM) supplemented with 10 mM uracil. Strain G809, the Δ*yapA* mutant (Δ*ligD pyrG*^-^ Δ*yapA*::*niaD pyrG*^+^), and the complemented strain (Δ*ligD pyrG*^-^ Δ*yapA*::*yapA niaD pyrG*^+^) were cultured on ANM for 10 d. The conidia of G809, mutant, and complemented strain were collected by cotton swab-scraping followed by resuspension in sterile normal saline containing 0.05% Tween 40. The suspension was filtered through sterile glass wool to harvest for the conidia.

### Gene deletion and complementation

To investigate the role of *yapA* in *T*. *marneffei*, the Δ*yapA* mutant was constructed according to homologous recombination method described by Borneman and collaborators [19]. Briefly, the transformed plasmid was constructed beginning with PCR amplification of the *yapA* gene. The YapAF and YapAR primers (Table 1) were used to amplify the *yapA* gene consisting of 2 kb upstream and downstream untranslated regions (UTRs) from *T*. *marneffei* G809 genomic DNA. The 7-kb purified product was cloned into PTG-19T PCR cloning vector (Vivantis, Malaysia) to generate a pWyapA plasmid. The *yapA* deletion construct was generated by using pWyapA plasmid as a template in an inverse PCR reaction for amplification of the 5’ and 3’ flanking region of the *yapA gene* and PTG-19T plasmid using a Phusion^®^ Hot Start Hight Fidelity DNA Polymerase (Thermo Fisher Scientific, Espoo, Finland). The inverse PCR product was digested with *Cal*I and *Not*I. After purification, the PCR product was ligated into *Cal*I/*Not*I *pyrG* (*Aspergillus nidulans*) blaster selectable marker cassette (pAB4626) [19] by site-specific digestion to generate the pDyapA plasmid (S1 Fig). Subsequently, the plasmid pDyapA was transformed into *Escherichia coli* DH5α and isolated. A linear fragment of gene deletion construct plasmid was generated by using PCR with YapAF and YapAR primers, and it was used for transformation into *T*. *marneffei* protoplasts. The sequences of primers used in this study are shown in Table 1.

**Table 1:** Primers sequences used for deletion and complementation of *yapA* gene in this study.

Primer	5’-3’ sequence
YapAF	**5’** CTACTACAGTAGAGCCTTGC **3’**
YapAR	**5’** GACAGGTGGATGAGAGTGC **3’**
NotI-SEF	**5’**TAGCGGCCGCGAGACCGTGTTAGTTGAG **3’**
CalI-SER	**5’** CCATCGAT CCTCATGCTTCCTTGTTC **3’**
NotI-yapAF	**5’**TAGCGGCCGCGCTACTACAGTAGAGCCTTGC**3’**
NotI-yapAR	**5’**ATGCGGCCGCGACAGGTGGATGAGAGTGC **3’**

To construct the complemented strain, the PCR product of the *yapA* gene was amplified using *Not*I-yapAF and *Not*I-yapAR primers, then digested with *Not*I restriction enzyme. The *Not*I fragment from the PCR product of the *yapA* gene was ligated into *Not*I-digested and dephosphorylated pAB4626 vector, yielding the pCyapA (S1 Fig). The plasmid pCyapA was then transformed in *Escherichia coli* DH5α and isolated. The primers are described in Table 1. 5-fluoorotic acid was used to select the spontaneous *pyrG* mutant (uracil auxotroph) from the *yapA* deletion mutant strain, and then the mutant was complemented by the pCyapA vector containing the *yapA* gene plus the selectable marker (*pyrG*) gene.

After transformation, the protoplasts were cultured on protoplast medium supplemented with 1.2 M sorbitol. The plates were incubated at 25°C for 5 d and then transferred to 37°C for 5 d. Southern blot hybridization on a selected transformant was performed to demonstrate the homologous integration at the target *T*. *marneffei* locus. The DNA probe was labeled with alkaline phosphatase enzyme using the gene images AlkPhos direct labelling and detection system (GE Healthcare, UK).

### Protoplast preparation

*T*. *marneffei* uracil auxotroph strain G816 (Δ*ligD niaD pyrG*^-^) was cultured in synthetic dextrose (SD) medium supplemented with 5 mM uracil. The culture flask was shaken in an incubator at 150 rpm for 3 d at 25°C. Fungal cells were filtered through Miracloth (Calbiochem, Germany) and then washed with 0.6 M MgSO_4_. Two scoops of mycelia were transferred into flask containing 10 ml of Osmotic medium (OSMO; 1.2 M MgSO_4_, 10 mM sodium phosphate, pH 7.0), 100 mg lytic enzyme (from *Trichoderma harzianum*, Sigma-Aldrich, UK) and 12 mg bovine serum albumin (BSA, Sigma-Aldrich, UK). The flask was then incubated in a shaking incubator at 37°C for 1.5 h. Cells were transferred into 50 ml centrifuge tubes. OSMO was added at equal volume and then layered with 10 ml of trapping buffer (trapping buffer; 0.6 M D-Sorbitol, 100 mM Tris-HCl pH 7.0) on the top. The tubes were centrifuged at 4,500 rpm for 20 min. Protoplasts that accumulated in a white band appearing at liquid-liquid interface were collected into a new tube and centrifuged at 4,500 rpm for 20 min. The resulting pellet was suspended with 5 ml of Sorbitol, Tris, CaCl_2_ (STC) buffer and centrifuged at 7,000 rpm for 5 min. 300 μl of STC was added to resuspend the pellet.

### Transformation and transformant selection

Based on chemically-induced transformation, protoplasts were treated with 60% polyethylene glycol (PEG) 4000 [19]. The 100 μl of protoplast suspension was added into 2 tubes (DNA+ and DNA- control). The linear fragment of gene deletion construct (about 500 ng) was added into DNA+ tube. The 25 μl of 60% PEG 4000 was added and then mixed by pipetting. The transformation tubes were incubated on ice at least 30 min. 1 ml of 60% PEG was added and rolled slowly to mix. Next, these tubes were incubated at room temperature for 30 min. Subsequently, 5 ml of STC were added into each tube and all tubes were spun at 4,500 rpm for 20 min. The pellet was suspended in 300 μl of STC. After the transformation, the protoplasts were plated on protoplast medium with 1.2 M sucrose (Bio basic Canada INC., Canada). The plates were incubated at 25°C for 12 d.

### Morphology and growth study of *the ΔyapA* deletion mutant

Macroscopic morphologies of *T*. *marneffei* G809, the *yapA* mutant, and the complemented strain were observed. Each strain was cultured on ANM agar at 25°C for 12 d and on brain heart infusion agar (BHA, Difco) at 37°C for 12 d, to generate the mold and yeast phase, respectively. The macroscopic morphologies of mold and yeast forms were visualized using the stereomicroscope (Drawell, China). The microscopic morphology of the mold form was examined under light microscopy (Nikon Eclipse 50i, Tokyo, Japan) using a slide-culture technique on ANM incubated for 12 d at 25°C. To observe yeast cells in liquid medium, conidia were incubated in 1% peptone broth at 37°C, shaking at 150 rpm for 12 d. The morphology was observed under light microscopy and photographed.

For staining with Calcofluor White (CFW) (fluorescent brightener 28, Sigma- Aldrich), fixed slide-cultures of the mycelial form were stained with a 0.1% (w/v) solution of the fluorescent dye at room temperature for at least 5 min. For yeast cells, the samples were collected, smeared on slide and stained with a 0.1% (w/v) solution of the CFW at room temperature for at least 5 min. Then, samples were examined under fluorescent microscopy (Nikon Eclipse 50i, Tokyo, Japan).

To understand the role of *yapA* in growth and conidiation, 10^6^ conidia of each strain were spotted on ANM and incubated at 25°C for 12 d. The diameters of ten individual colonies were examined every day for up to 12 d. The conidia counts were examined at day 12. The conidia were harvested by cotton swab-scraping and resuspension of the collected conidia in sterile normal saline containing Tween 40.

For germination assays, 10^6^ conidia of each strain were inoculated in 300 μl of Sabouraud dextrose broth (SDB, Difco) and incubated at 25°C or 37°C with continuous shaking at 150 rpm. The presence of germinating cells were observed every 3 h for a maximum of 24 h. Mean and standard deviation (SD) values were calculated and analyzed for statistical significance using GraphPad Prism 5 program.

### Investigation of oxidative stress susceptibility of *yapA* mutants

A conidial suspension was serially diluted and 5 μl drops of each dilution (10^5^–10 cells/μl) were spotted onto ANM medium or BHA medium containing various stressors at different concentrations. Menadione and H_2_O_2_ were used as the oxidative stressors and NaNO_2_ was used for induction of nitrosative stress. The concentrations of stressors were as follows: 0.05 mM and 0.1 mM menadione; 0.5 mM and 1 mM H_2_O_2_; and 6 mM and 12 mM NaNO_2_. ANM or BHA media without stressor were used as controls. The ANM medium was used to grow the mycelial phase, whereas BHA medium was used to grow yeast phase. Plates were incubated at 25°C or 37°C for ANM and BHA, respectively. Growth of the wild-type (G809), mutant, and complementary strains were examined after 10 d of incubation.

### Investigation of *yapA* function against antifungal activity in a human macrophage (THP-1) cell line, *in-vitro*

The THP-1 human monocytes (THP1, American Type Culture Collection), kindly provided by Prof. Patrick Woo (Department of Microbiology, The University of Hong Kong, Hong Kong, China), were cultured in RPMI 1640 medium (GIBCO-BRL, Life Technologies) supplemented with 10% fetal bovine serum at 37°C and 5% CO_2_. To induce monocyte to macrophage differentiation, THP-1 cells were seeded into 24-wells tissue culture plated at 1X 10^5^ cells per well, containing 100 ng/ml phorbol 12-myristate 13-acetate (PMA, Sigma) and incubated at 37°C, with 5% CO_2_ for 48 h. The culture medium was removed and 1 ml RPMI medium containing 5 X 10^5^ conidia (MOI = 5) was added to each well (adapted from Nimmanee *et al*., 2015). To study the survival of *T*. *marneffei*, the THP-1 cells were incubated with the conidia for 2 h to allow adhesion and phagocytosis. After 2 h, each well was washed with RPMI medium with 240 U/ml of nystatin (Sigma—Aldrich, St. Louis, USA) to kill extracellular conidia. Thereafter, the nystatin was removed and replaced with fresh RPMI, and then THP-1 cells were incubated for 12 h and 24 h. After incubation, 1% Triton X-100 (Sigma—Aldrich, St. Louis, USA) was added to lyse the infected macrophages. The cell lysate was diluted, spread on PDA and incubated at 25°C for colony forming unit (CFU) count. The experiments were performed in triplicate independent tests. The statistical significance was calculated and analyzed using GraphPad Prism 5 program.

## Results

### Characterization of *T*. *marneffei* bZIP transcription factor AP-1/Yap1

**Fig 1:**
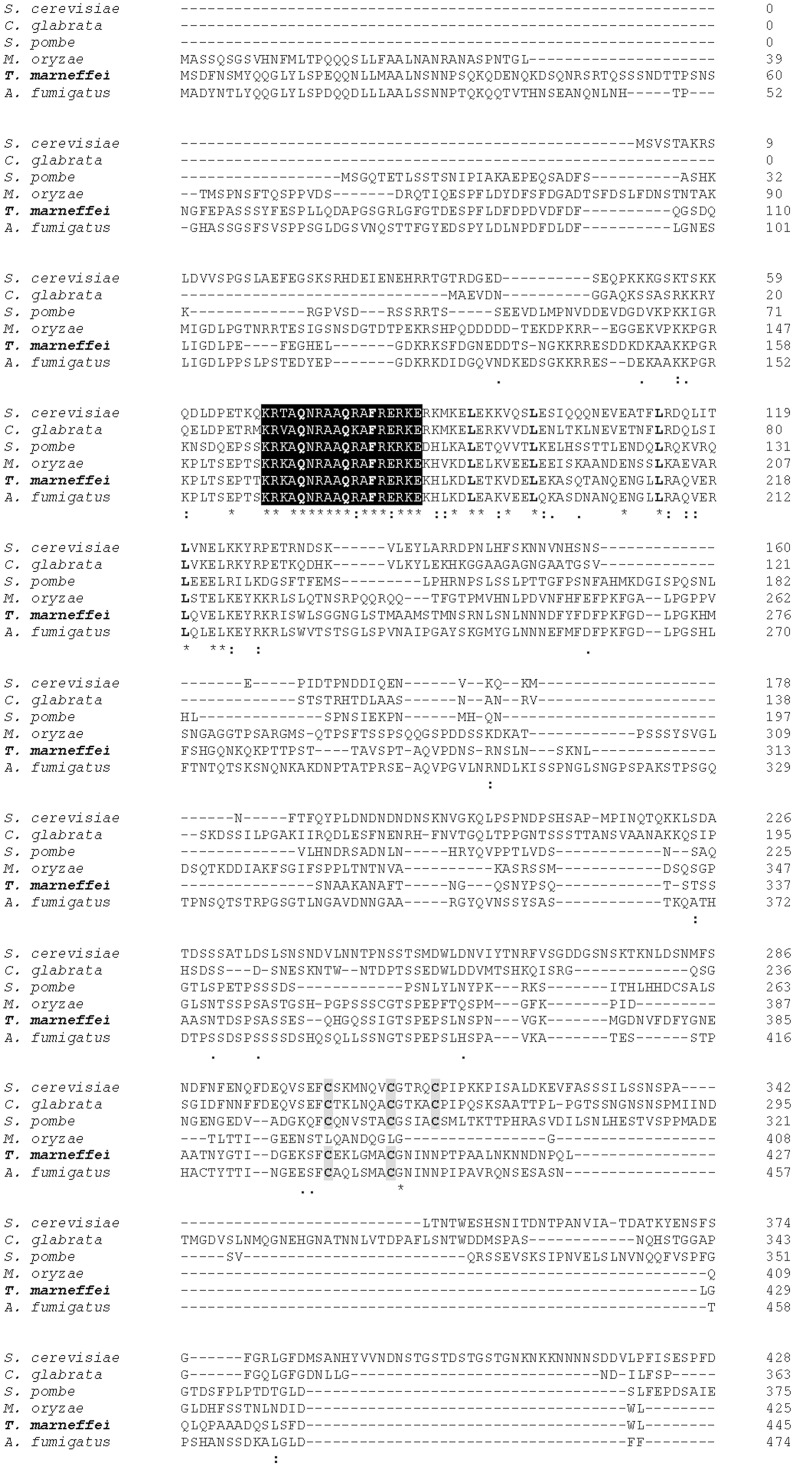
Protein sequence alignment of the bZIP transcription factor AP-1/Yap1 homologs from *S*. *cerevisiae* (gi|4798|emb|CAA41536.1), *C*. *glabrata* (gi|49526305|emb|CAG59929.1), *S*. *pombe* (gi|5001|emb|CAA40363.1), *M*. *oryzae* (gi|145608602|ref|XP_001408783.1), *T*. *marneffei* (gi|212531151|ref|XP_002145732.1), and *A*. *fumigatus* (gi|70992066|ref|XM_745789.1). The domains important for function are indicated as follows: the bZIP_Yap region is shaded black; cysteine amino acids are shaded grey in the N-terminal cysteine rich domain and the C-terminal cysteine rich domain.

The *yapA* coding sequence of *T*. *marneffei* (accession number XP_002145732.1) with a length of 2,947 bp was predicted to encode a polypeptide of 592 amino acids. A multiple amino acid alignment of *T*. *marneffei* bZIP transcription factor AP-1/Yap1, designated YapAp, using Clustal Omega program (http://www.ebi.ac.uk/Tools/msa/clustalo/), showed that the *T*. *marneffei* YapAp shared 26.07% identity with Yap1P from *S*. *cerevisiae*, 25.99% identity with hypothetical protein from *Candida glabrata*, 25.92% identity with Pap1p from *S*. *pombe*, 38.51% identity with hypothetical protein MGG_12814 from *Magnaporthe oryzae*, and 56.08% identity with Yap1 from *A*. *fumigatus*. The YapAp of *T*. *marneffei* shared several conserved domains with various yeast and filamentous fungi, including a bZIP_YAP domain, and two cysteine-rich domains, n-CRD and c-CRD, which are N-terminal to C-terminal, respectively. The highly conserved sequences are at the bZIP domain. At n-CRDs from bZIP transcription factor AP-1/Yap homologs of various yeast and filamentous fungi, differences were identified in these amino acid sequences (Fig 1).

### Selection of the *yapA* gene deletion mutant and complemented strain

**Fig 2:**
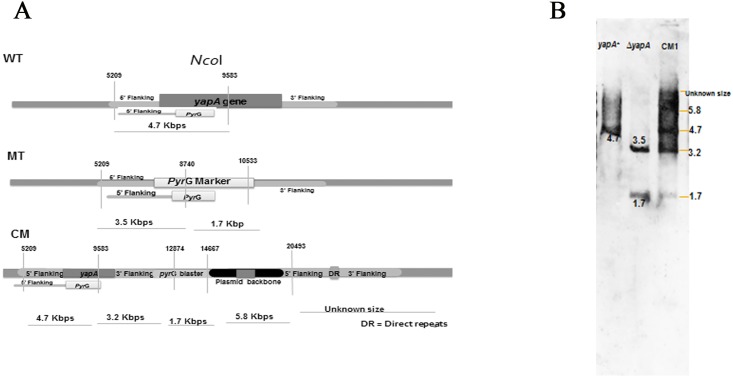
Southern blot analysis of the wild-type, mutant, and complemented strains. Schematic representation of the genomic *yapA* locus of *T*. *marneffei*, *Nco*I cleavage sites, and the position of the probe hybridization are indicated (A). The chromosomal DNA of wild type (lane 1), mutant (lane 2), and complemented strain (lane 3) was digested with *Nco*I. The 2.5 kb containing the 5’ UTR of *yapA* gene and *pyrG* marker was used as a probe. The bands characteristic of wild type, mutant, and complemented strain displayed the expected size differences (B).

To study the role of the *yapA* gene in *T*. *marneffei*, the *yapA* deletion mutant was constructed by replacing the open reading frame (ORF) of the *yapA* gene with the *pyrG* selectable marker. The transformants, which were grown on selection media, were checked for the absence of *yapA* by PCR using gene specific primers (data not shown). Further, the role of *yapA* gene was confirmed by gene complementation. The complemented strains that showed colonies similar to the wild type (G809) were checked for the *yapA* integration using PCR (data not shown). The copy number of the transforming vector and its integration site in the genome of the mutant and complemented strain was ascertained by Southern blot hybridization. The genomic DNA of wild-type (G809, *yapA*^+^), mutant (*ΔyapA*), and complemented (CM1) strains was extracted, digested with *Nco*I restriction enzyme, then hybridized with the DNA probe containing the 5’ UTR of *yapA* gene and *pyrG* marker. The schematic representation of the genomic *yapA* locus of *T*. *marneffei* is represented in the Fig 2a. The bands in the x-ray film were shown as expected for the single copy gene integration (Fig 2B). Only a single band of about 4.7 kb was detected in the wild type. Two bands of approximately 1.7 and 3.5 kb were detected in the mutant. Four expected bands were seen in the complemented strain CM1. These data indicated that both of the mutant and CM1 possessed a single copy of the transforming vector in the genome. The mutant and complemented strain were used in further studies.

### Morphology and growth study of the *ΔyapA* deletion mutant

**Fig 3:**
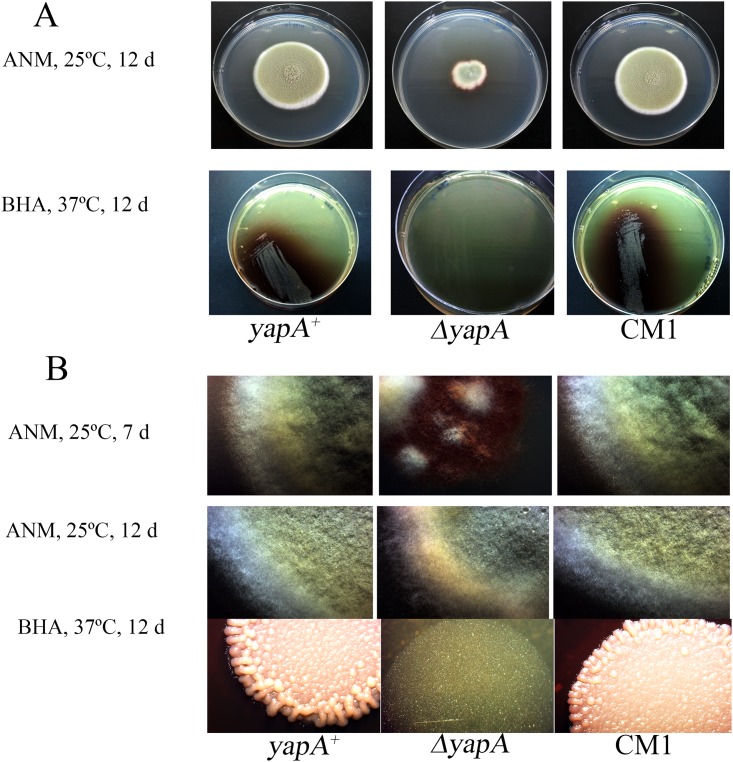
The macroscopic examinations of *T*. *marneffei* wild type G809, Δ*yapA* mutant, and complemented strain. The mold and yeast form were examined by optical visualization, the mold phase was represented on the top and yeast on the bottom, respectively (A). The stereomicroscope examination of the mold colonies at day 7 and 12, and yeast at day 12 (B).

**Fig 4:**
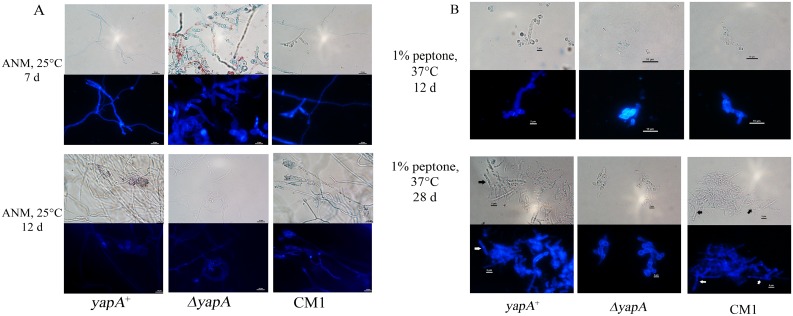
The microscopic examinations of mold phase using a slide culture technique (A). Microscopic examinations of the yeast phase from the 1% peptone culture broth (B). Both mold and yeast phase were stained with calcofluor white to visualize chitin deposition in cell walls and septa.

On ANM media at 25°C, the wild type and complemented strain displayed similar growth of powdery to velvety colonies with yellowish-green color, whereas the *yapA* mutant had slow growth rate showing smaller colony with more red pigment production than those of the wild type and complemented strain (Fig 3A), including fewer hyphae and conidia formation observed under stereomicroscope at day 7 (Fig 3B). However, at day 12, the mutant could produce the powdery to velvety colony with yellowish-green like wild type and complemented strain but smaller colony diameters than wild type and complemented strain (Fig 3b lower panel). Microscopically, wild type and complemented strain showed septate, hyaline hyphae with conidiophore and metulae bearing 4 to 7 phialides (Fig 4A). Each metulae produced long chain of ellipsoidal-shaped conidia, whereas fewer phialides and conidia were seen in the mutant. At day 12, the mutant could produce conidia which the morphology similar to the wild type and complemented strain, however, the colony numbers of mutant less than wild type and complemented strain (Fig 4A). These observations indicate that the *yapA* may have a role in normal hyphal development and pigmentation in *T*. *marneffei*.

When incubated at 37°C for 12 d, the yeast colony of the wild type and the complemented strain appeared glabrous and beige in color with brown pigment. In contrast, only a small colony without brown pigment was observed in the mutant (Fig 3A and 3B). Microscopically, from a 1% peptone broth culture incubated at 37°C for 12 d, the wild type and complemented strain showed yeast-like organisms 3 to 8 microns in length with a characteristic elongate yeast cells with internal fission. By comparison the mutant strain could not divide by fission (Fig 4B top). From this data, we investigated whether *yapA* involved the processing of binary fission in *T*. *marneffei*. The mutant was incubated in 1% peptone at 37°C for 28 d, then observed under microscope. Surprisingly, the mutant displayed swollen cells and no internal fission in their cells (Fig 4B lower panel). These data suggest that the *T*. *marneffei yapA* gene is involved in binary fission.

**Fig 5:**
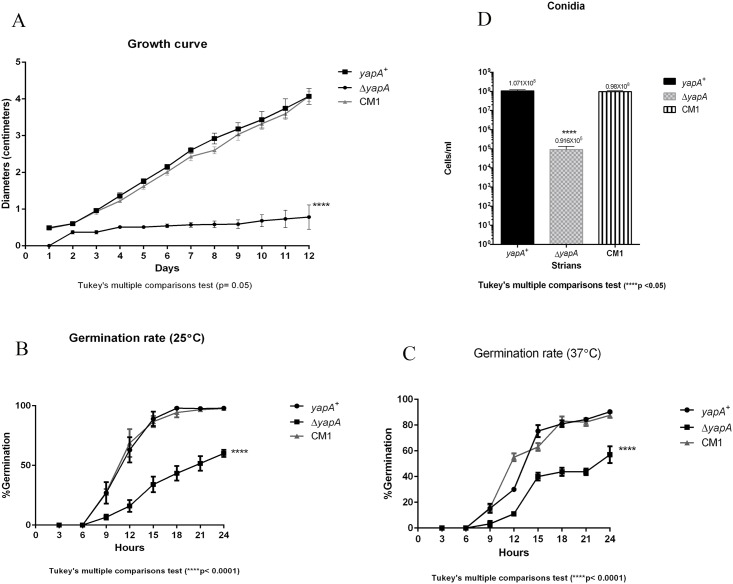
Growth studies of the *ΔyapA* deletion mutant. The growth curves (radial diameter) of *T*. *marneffei* wild type, *ΔyapA* mutant, and complemented strain grown in ANM medium for 1 to 12 d at 25°C (****, p value < 0.05) (A). The germination rates of wild type, *ΔyapA* mutant, and complemented strain which grown in SDB medium for 24 h at 25°C (****, p value < 0.001) (B) at 37°C (****, p value < 0.001) (C). The conidia number of wild type, *ΔyapA* mutant, and complemented strain cultured on ANM medium (****, p value < 0.05) (D).

Our macroscopic examinations suggested that the growth rate of the wild-type and complemented strains were similar, but that of the mutant strain was dramatically delayed. The colony diameter of mutant was approximately 1 cm, whereas colonies of the wild type and complemented strain were about 4 cm in diameter after 12 d of incubation at 25°C (Fig 5A). This suggested that the *yapA* mutant may have a defect in radial growth.

Under suitable culture conditions, dormant conidia germinate to produce the hyphal form at 25°C or the yeast cells at 37°C. Given the possible role of the *yapA* gene in morphogenesis, we studied the germination rates of conidia both at 25°C and 37°C. The results showed significantly slower germination of the mutant compared to the wild type and complemented strain (Fig 5B and 5C). In addition, we examined the number of conidia produced by these three strains at day 12 of incubation at 25°C on ANM medium. These results indicated that the mutant produced less conidia than the wild type and complemented strain (Fig 5D). Hence, the *yapA* gene appears to be involved in morphogenesis, development, and conidiation in *T*. *marneffei*.

### The *yapA* disruption strain exhibits decreased oxidative tolerance

**Fig 6:**
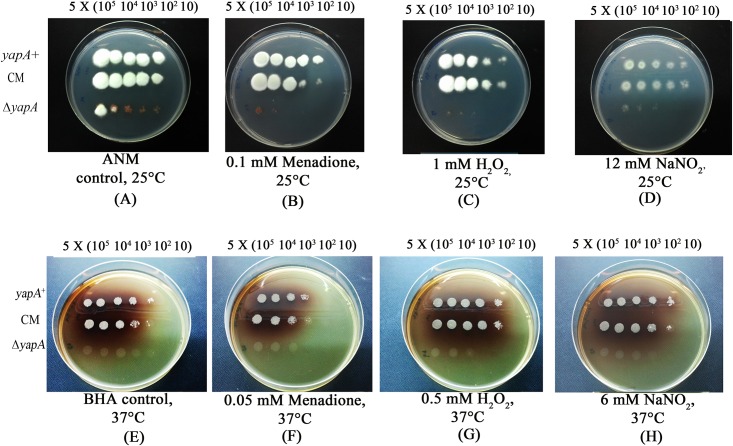
The *T. marneffei ΔyapA* mutant is more sensitive to oxidative and nitrosative stress. The wild type, mutant, and complemented strain were grown for 7 day in ANM agar at 25°C (A), ANM plus 0.1 mM Menadione (B), ANM plus 1 mM H_2_O_2_ (C), ANM plus 12 mM NaNO_2_ (D), BHA agar at 37°C (E), BHA plus 0.05 mM Menadione (F), BHA plus 0.5 mM H_2_O_2_ (G), and BHA plus 6 mM NaNO_2_ (H).

The Yap1 in *S*. *cerevisiae* plays a role oxidative stress response. To determine the function of the *T*. *marneffei yapA* gene in stress tolerance, the wild type, mutant, and complemented strains were exposed to H_2_O_2_, menadione, and NaNO_2_. The mycelial growth of the mutant was hypersensitive to 0.1 mM menadione, and 1 mM H_2_O_2_ when compared with the wild-type and complemented strains. In addition, the mutant was also more susceptible to 12 mM NaNO_2_ than the wild type and complemented strain. Moreover, the yeast phase of the mutant strain was more sensitive to 0.05 mM menadione, 0.5 mM H_2_O_2_, and 6 mM NaNO_2_ than the wild type and the complemented strain (Fig 6).

### The *yapA* disruption strain exhibits decreased survival in a human macrophage THP-1

**Fig 7:**
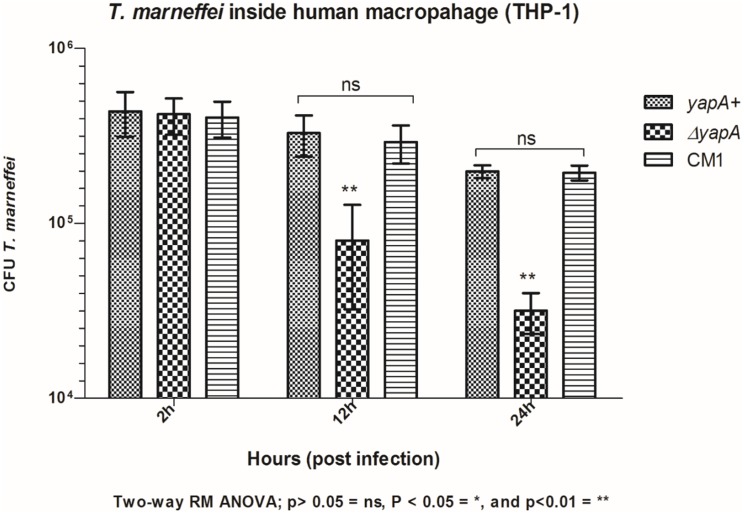
Antifungal activity of THP-1 macrophage. The macrophages were infected with the conidia at 5 MOI for 2 h, then removed the excess conidia and continuously incubated for 12 h and 24 h followed by cell lysis and *T*. *marneffei* cell counting. The data are expressed as mean CFU ± SEM recovered from triplicate cultures.

To assess whether *T*. *marneffei yapA* is involved in response against the antifungal activity of macrophages, human THP-1 macrophages were infected *in vitro* with *T*. *marneffei* conidia for 2 h. Infected macrophages were subsequently incubated for 12 to 24 h followed by cell lysis and the counting of viable *T*. *marneffei* in terms of colony forming units (CFU). At 2 h, the baseline, the CFU of the wild type was 4.3x10^5^ cells/ml, the mutant was 4.2x10^5^ cells/ml, and CM1 was 4.1x10^5^ cells/ml. After 12 h post infection, the CFU of mutant decreased significantly (8x10^4^ cells/ml) when compared to the wild type (3.2x10^5^ cells/ml) and complemented strain (2.9x10^5^ cells/ml). Moreover, at 24 h post infection, the CFU of the mutant reduced significantly (3.2x10^4^ cells/ml) more than wide type (2x10^5^ cells/ml) and complemented strain (1.8x10^5^ cells/ml) (Fig 7). Therefore, it appears that *yapA* plays a significant role in intracellular survival.

## Discussion

The aim of this investigation was to study the function of the *T*. *marneffei yapA* gene that encoded a putative bZIP transcription factor protein, based upon homology to other similar fungal genes. The *yapA* deletion mutant was generated using homologous recombination principle in a strain deficient in non-homologous end joining (G816) [18]. To confirm that the mutant phenotype was due to the *yapA* function, the entire *yapA* sequence was transformed into the mutant to generate a complemented strain.

The *T*. *marneffei* YapAp belongs to the fungal AP-1 proteins, similar to those of *S*. *cerevisiae* Yap1p and *S*. *pombe* Pap1p. By comparing amino acid sequences common to the AP-1 family, the *T*. *marneffei* YapAp contains a bZIP_Yap domain at the N-terminal, and two cysteine rich domains including, n-CRD and c-CRD domains that contain the nuclear export signal (NES). These domains are critical for regulating the Yap1p localization [12]. The Yap1 protein is conserved in many fungi and functions in regulating oxidative stress responses. In dimorphic fungi, it has been reported that the YAP1 homolog was found in the *Paracoccidioides brasiliensis* transcriptome, but its function has yet to be reported [20]. However, the role of Yap1 in virulence differs subtly among various fungal pathogens.

This study demonstrated for the first time the role of a *yap1* ortholog in a dimorphic fungus, *T*. *marneffei*. Interestingly, the *T*. *marneffei* Δ*yapA* mutant showed a redial growth defect and also developmental deficiencies. The mutant produced fewer hyphae and increased red pigment production at 25°C when culture for 7 d (Fig 3B). This result was a surprising, raising the question of whether *yapA* is involved red pigment production. Recently, the red pigment, which is produced by *T*. *marneffei* (mold phase), was investigated. In 2007, Bhardwaj and colleagues purified the red pigment and investigated the putative structure, which was claimed to have a structure similar to herqueinone and contains a phenalene carbon framework. Red pigment production in *T*. *marneffei* is regulated by polyketide synthase (PKS) genes, such as the genes encoding a polyketide synthase (PKS3), fatty acid synthases and other genes [21]. Conceivably, the *yapA* gene may play a role in regulation of this gene cluster. Curiously, after incubating for 12 d at 25°C, the *ΔyapA* mutant of *T*. *marneffei* exhibited a colony diameter of approximately 1 cm, whereas colonies of the wild type and complemented strain were about 4 cm (Fig 5A). However, no defect in aerial hyphal growth of the mutant was observed at day 12 (Fig 3B). The delay in growth resulted in reduced conidial formation. The defect of conidial formation was also reported in the *Moap1* (*yap1* ortholog) mutant of rice blast fungus, *Magnaporthe oryzae* [22]. In addition, the *T*. *marneffei* Δ*yapA* mutant displayed the delay in conidial germination similar to those seen in an *E*. *festucae* Δ*yapA* mutant [23]. On BHA at 37°C, the wild-type and complemented strains of *T*. *marneffei* displayed beige-colored yeast-like colonies with some brown pigment production, whereas the mutants produced small colonies without brown pigment. In *M*. *oryzae*, deletion of *Moap1* caused reduced pigmentation. Interestingly, the *Moap1* mutant had reduced the activity of the secreted laccase which is known to be involved in pigment production [22]. In addition, the *T*. *marneffei ΔyapA* strain was unable to undergo fission development when cultured in 1% peptone broth at 37°C within the time of investigation (28 d) (Fig 5B). Hence, the *yapA* gene also appears to be involved cellular division of the yeast phase. Alternatively, the severe growth delay in yeast phase might be explained by heat shock response defect.

The Yap1 protein is conserved in many fungi and functions in the regulation of oxidative stress response, growth, conidia formation, and the production of peroxidases, laccases and melanin. However, the role of Yap1 in virulence is quite diverse. The role of Yap1p in the regulation of enzymes that protect against oxidative stress was first suggested when the *yap1* deletion mutant (*Δyap1*) in *S*. *cerevisiae* was found to be hypersensitive to H_2_O_2_ and t-butyl hydroperoxide (t-BOOH) as well as chemical reagents that generate superoxide anion radical (menadione, methylviologen and plumbagine) [13]. In *S*. *pombe*, the transcription factor Pap1p regulated gene expression in response to the oxidase stress [15]. In *C*. *albicans*, loss of the Cap1 function leaded to reduced tolerance to the oxidative stress and drug stress [16, 24]. For the pathogenic fungus, *A*. *fumigatus*, the deletion of *Afyap1* gene led to hypersensitivity to stressors such as H_2_O_2_ and menadione. However, AfYap1 does not appear to be involved in the pathogenicity of *A*. *fumigatus* [17]. The role of Yap1 has been reported in plant pathogenic fungi, such as *Ustilago maydis* Yap1 [25], and *Alternaria alternata* AaAP1 [26]. Moreover, the Moap1 mutant was sensitive to H_2_O_2_ and displayed several defects in aerial hyphal growth, branching, conidia formation, as well as the production of peroxidases, laccases and melanin. However, Moap1 was not required for infection in the host plant [22]. In the present study, we noted that the *T*. *marneffei yapA* gene is involved in the oxidative stress response similar to other fungi. Moreover, the *T*. *marneffei yapA* gene appears to have a role in nitrosative stress tolerance with the *yapA* deletion mutant being sensitive to NaNO_2_. Interestingly, the growth of mutant was inhibited by the human macrophage cell line, THP-1. Therefore, in *T*. *marneffei*, the *yapA* gene may be involved in tolerance to reactive oxygen species which are produced by macrophage. This response is similar to that reported for the transcription factors *sakA* and *atfA* [27, 28]. Based on our results, we suggest that *yapA* in *T*. *marneffei* is involved in oxidative tolerance as well as serving as a defense mechanism against macrophage attack.

Finally, a number of genes that are transcriptionally regulated by the *S*. *cerevisiae* Yap1p have been identified. The glutamylcysteine synthetase *(gsh1*) gene is one of the several genes that are transcriptionally regulated by Yap1p. A site-directed mutation in the YRE of *gsh1* blocks Yap1 binding to *gsh1*. A mutant lacking *yap1* is hypersensitive to methylglyoxal as the same as cells lacking the *gsh1* gene [29]. In addition other glutathione genes, expression of the glutathione reductase (*glr1*), glutathione peroxidase (*gpx2*) and glutathione synthetase (*gsh2*) genes are dependent upon the Yap1 transcriptional activator protein [30]. The *S*. *pombe* Δ*pap1* mutant is also unable to promote expression of *trx2*, *trr1* (thioredoxin reductase encoding gene), *ctt1* (catalase encoding gene) in response to H_2_O_2_ [31]. Furthermore, *Afyap1* gene in *A*. *fumigatus* regulates several defense genes against oxidative stress such as catalase gene, thioredoxin reductase, and other anti-oxidant genes [17]. Therefore, future studies in identification of the genes that are transcriptionally regulated by *yapA* in *T*. *marneffei* would provide a better understanding of oxidative and stresses responses of this fungus as well as help detail signaling pathways.

## Supporting Information

S1 Fig The strategies to study the role of *yapA* gene. The construction of pDyapA deletion plasmid used for deleting the *yapA* gene in *T*. *marneffei* (A). The generation of pCyapA plasmid to complement the *yapA* gene into the mutant strain (B).
(TIF)
