# Supplementary figures and images for: The yapA Encodes bZIP Transcription Factor Involved in Stress Tolerance in Pathogenic Fungus Talaromyces marneffei
Source: PLoS One. 2016 Oct 5;11(10):e0163778. doi: 10.1371/journal.pone.0163778 (PMC5051730; doi:10.1371/journal.pone.0163778)

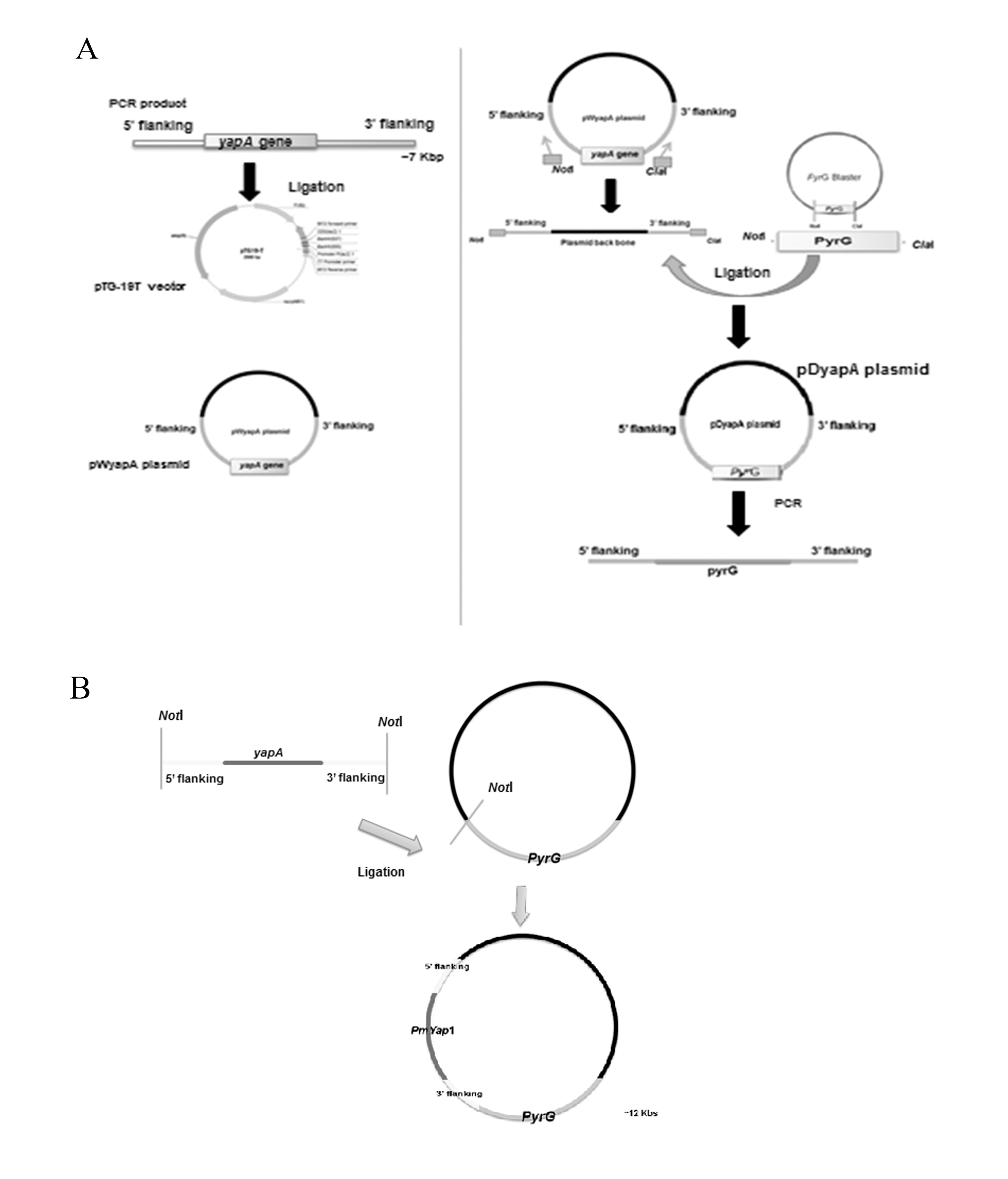

Supplement: S1 Fig — The construction of pDyapA deletion plasmid used for deleting the yapA gene in T. marneffei (A). The generation of pCyapA plasmid to complement the yapA gene into the mutant strain (B). (TIF) [file pone.0163778.s001.tif]
